# Study protocol for Post Pregnancy Family Planning Choices, an operations research study examining the effectiveness of interventions in the public and private sectors in Indonesia and Kenya

**DOI:** 10.12688/gatesopenres.13147.2

**Published:** 2020-12-18

**Authors:** Elaine Charurat, Sara Kennedy, Siti Qomariyah, Anne Schuster, Megan Christofield, Lindsay Breithaupt, Emmah Kariuki, Michael Muthamia, Mark Kabue, Eunice Omanga, Jelle Stekelenburg

**Affiliations:** 1 Jhpiego, Baltimore, Maryland, USA; 2 Jhpiego, Jakarta, Indonesia; 3 Jhpiego, Nairobi, Kenya; 4 University of Groningen, Groningen, The Netherlands

**Keywords:** postpartum family planning, postabortion family planning, study protocol

## Abstract

**Background: **Global evidence suggests many postpartum and postabortion women have an unmet need for family planning (FP) after delivery or receiving care following loss of a pregnancy. Post Pregnancy Family Planning Choices, an operations research study, aims to examine the effectiveness of a package of postpregnancy FP interventions, inclusive of postpartum and postabortion FP. The interventions are being implemented in selected public and private facilities in Indonesia and Kenya and focus on quality FP counseling and service provision prior to discharge. This manuscript presents the study protocol, documenting how the study team intends to determine key factors that influence uptake of postpregnancy FP.

**Methods: **This is a multi-country, quasi-experimental three-year operations research study in Brebes and Batang Districts of Indonesia and Meru and Kilifi Counties of Kenya. Quantitative and qualitative data is collected longitudinally through interviews and health facility assessments at multiple time points. Data is gathered from 22 health facilities; 8,796 antenatal, postpartum, and postabortion clients; and key informants at national, subnational, facility, and community levels. Quantitative study data is collected and managed using REDCap (Research Electronic Data Capture). Once data are thoroughly cleaned and reviewed, regression models and multilevel analyses will explore quantitative data. Qualitative study data is collected using audio recordings and transcribed to Microsoft Word, then analyzed using ATLAS.ti. Qualitative datasets will be analyzed using grounded theory methods.

**Discussion: **The ultimate goals of the study are to generate and disseminate actionable evidence of positive drivers, barriers, and activities that do not yield results with regard to increasing postpregnancy FP programmatic activities, and to institutionalize postpregnancy FP in the public and private sectors in Indonesia and Kenya. We hope these learnings and experience will contribute to global efforts to advance and scale up postpregnancy FP in similar settings beyond these two countries.

**Trial registration: **ClinicalTrials.gov NCT03333473

## Introduction

Family planning (FP) is lifesaving, especially during the period immediately following childbirth. For the purpose of this study, we define postpregnancy clients as women who recently gave birth or experienced loss of pregnancy, commonly referred to as postabortion in African and Asian countries. Spacing pregnancies at least two years apart after a live birth not only prevents unintended pregnancies, but also lowers newborn, infant, and child mortality in subsequent pregnancies ^1^. World Health Organization (WHO) recommends spacing pregnancies by two years or more following the delivery of a newborn, and at least six months after receiving postabortion care ^2^. In 2015, WHO released its updated medical eligibility criteria allowing implants to be provided immediately after delivery ^3^, including among breastfeeding women, joining lactational amenorrhea, intrauterine devices (IUDs), and male and female sterilization, which were already approved for postpregnancy women by WHO. Since then, postpartum FP has received global and country-level attention as the result of the “Postpartum Family Planning Global Movement” through close collaboration and coordination among FP2020, WHO, donors, host-country governments, and implementers ^4^. While evidence collectively documenting successful programming experience around postpartum and postabortion FP has been widely disseminated ^5– 7^ for the public sector in many African and Asian countries, little is known about how feasible and effective it can be in the private sector, particularly among private-for-profit providers and facilities. Furthermore, postpregnancy women remain one of the most vulnerable groups with high unmet need for FP. Not only are there are major missed opportunities for FP among postpartum women in many low- and middle-income countries, but the majority of postabortion care clients still leave the facility without a contraceptive method ^7, 8^.

Prospective estimates find that 27% of Indonesian women and 63% of Kenyan women in their first-year postpartum have an unmet need for FP ^9, 10^. In Indonesia, while the contraceptive prevalence rate among all women of reproductive age is 47%, long-term methods such as implants, IUDs, and sterilization account for less than a quarter of contraceptives used, despite the higher effectiveness of these methods and the merits of a broader method mix ^11^. Data from Kenya demonstrates a great need for postpartum FP services, as 23% of births occur at intervals of less than 24 months, while only 19% of postpartum women begin using a FP method during the first six months postpartum and 36% between six and 12 months postpartum ^10^. For those who deliver at health facilities, 73% of women in Indonesia and 25% of women in Kenya deliver at a privately-owned facility as opposed to a public facility, pointing to a need to include private-for-profit facilities in the postpregnancy FP discussions ^9, 10^. Anecdotal evidence suggests FP uptake for women after receiving postabortion care is low but there is overall very little is documentation in either country at the time of study inception ^11^.

Based on unpublished findings, health care facilities where postpartum FP is introduced are also likely the places where postabortion FP may be needed, and should have already been introduced. Given that the service delivery platforms are similar and often the same for both, combining efforts at the facility level to cover broader postpregnancy FP for both postpartum and postabortion clients will conceivably allow these interventions to be carried out in a more coherent manner. More importantly, postpregnancy FP can reduce the burden of maternal and newborn mortality. If full provision of modern contraceptives were combined with adequate care for all pregnant women and newborns, maternal deaths in Asia and Africa combined would drop by 72–73% from 301,000 to 81,000 per year and newborn deaths would drop by 77-84% from 2.6 million to 0.5 million ^12, 13^.

Post Pregnancy Family Planning Choices (PPFP Choices) is an operations research study with intervention and control groups and a set of postpregnancy FP interventions that are inclusive of both postpartum and postabortion periods. PPFP Choices aims to generate actionable evidence to be used to increase programmatic activities to address postpregnancy FP in public and private sectors. We intend to work within existing public and private health facilities to strengthen the quality of postpregnancy FP counseling and service provision, which focuses on the prevention of unintended pregnancies through the first 12 months following childbirth and the first six months following loss of pregnancy.

**Figure 1.**
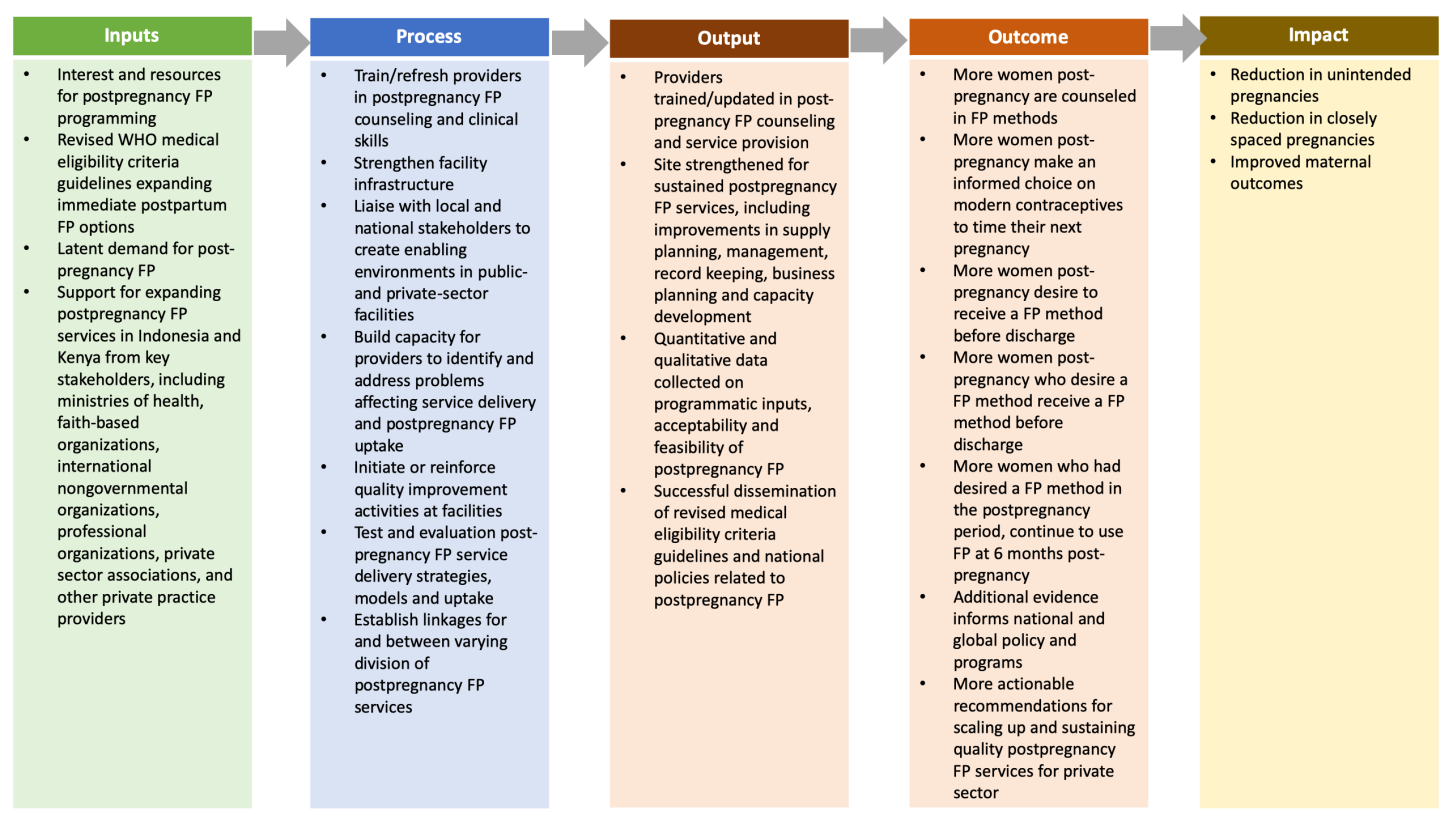
Post Pregnancy Family Planning Choices’ theory of change WHO, World Health Organization; FP, family planning.

The study follows a theory of change model ( Figure 1) with the intent to carry out a package of interventions to improve postpregnancy FP counseling and services in both public and private sectors. These interventions are designed and based on evidence and learnings from: 1) WHO’s *Programming Strategies for Postpartum Family Planning* ^5^; 2) private sector assessments in study sites conducted in 2017; 3) facility assessments focusing on opportunities of integrating postpartum and postabortion FP outside study sites conducted in 2016 by Jhpiego, prior to PPFP Choices’ inception; and 4) current and past programming experiences within and beyond Kenya and Indonesia. Broadly, PPFP Choices’ package of interventions includes:

- Capacity building in postpregnancy FP counseling and service provision during antenatal care (ANC), immediate postpartum, and immediate postabortion periods
- Quality improvement approaches to address system barriers at the facility level
- Private sector-specific interventions, such as business management skills strengthening
- Testing innovations in response to context-specific needs when settings permit

We use WHO’s building blocks for health systems to categorize the interventions within PPFP Choices:

1) *Health workforce:* providers are capacitated and equipped to provide quality postpregnancy FP counseling and service delivery during ANC, postpartum and postabortion periods through postpregnancy FP counseling and postpregnancy FP service provision, inclusive of postpregnancy IUD trainings.
2) *Service delivery:* building upon capacity building efforts, we employ relevant country-specific guidelines and standards to ensure quality service delivery.
3) *Health governance:* optimizing service efficiency, quality, and taking root of postpregnancy FP interventions within PPFP Choices is a key feature of the intervention package. In Indonesia, we follow existing quality assurance approaches/platforms as introduced by the Bill & Melinda Gates Foundation funded MyChoice (Right Method, Right Time, My Choice) project ^14^ and in Kenya, we use the Leadership Development Package plus ^15^.
4) *Health finance:* private sector-specific interventions are guided by private sector assessments conducted in both countries, focusing on barriers inhibiting private health facilities to offer quality postpregnancy FP counseling and services.
5) *Health information:* numbers of ANC visits, deliveries, postabortion care cases, postpregnancy FP counseling sessions, and uptake are captured in the facility registries, then summarized on a monthly basis. Note, these registers are used in health facilities in both intervention and control areas to compare indicators relevant to study interventions.
6) *Medical products:* we monitor health facilities in both intervention and control areas to make sure there are no stock-outs of contraceptive products and supplies that may hinder achievement of study outcomes and negatively affect the study environment. As needed, during the study period, we will correct a stock-out situation when it arises and supply or re-distribute when appropriate.

**Figure 2.**
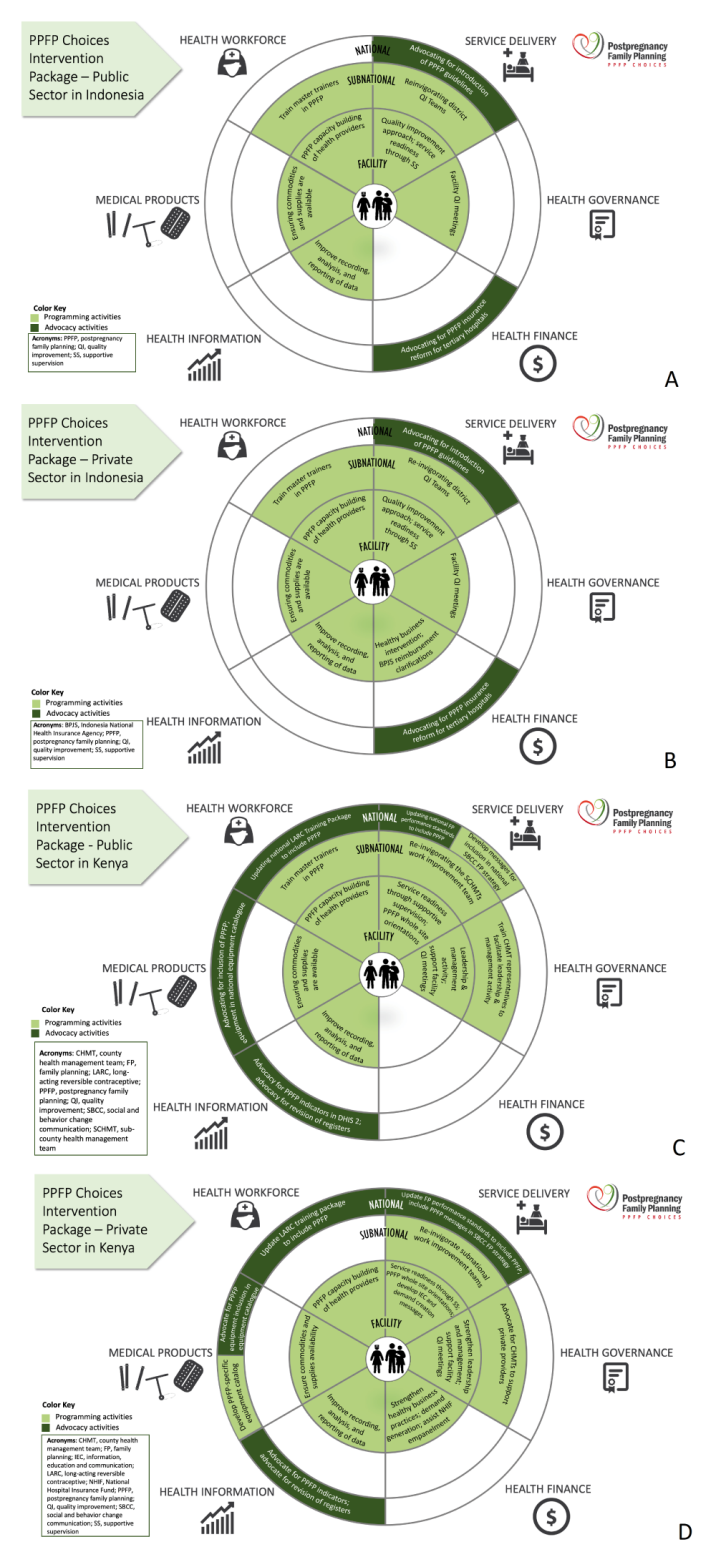
Post Pregnancy Family Planning Choices’ package of interventions for: **a**) public sector in Indonesia; **b**) private sector in Indonesia; **c**) public sector in Kenya; **d**) private sector in Kenya.

Country and sector specific interventions are designed to include activities at the facility, subnational, and national levels to encourage sustainable change across health systems. These are carried out as illustrated in Figure 2a–d. List of detailed activities implemented at the facility level can be found in the *Extended data* ^16^. 

PPFP Choices’ intervention package is being implemented in a phased manner. Beginning in late 2017, facilities in the intervention areas received the package of interventions prior to and throughout study activities in both countries. To ensure that control areas also benefit from study activities, the facilities in the control areas will receive the relevant and impactful aspects of the intervention package in 2020 after completion of all participant recruitment and data collection activities. In Indonesia, PPFP Choices is building upon and coordinating intervention activities with MyChoice project programming, while in Kenya, the intervention package is being introduced by Jhpiego in close collaboration with the Kenya Ministry of Health (MOH).

To respond to PPFP Choices’ mandate, our main research question is: “What are the key determinants at service delivery, provider, and client levels that influence the uptake of postpregnancy FP in the public and private health care sectors in Indonesia and Kenya?” as measured by FP uptake at the six-month postpregnancy period. We also have a series of secondary questions to evaluate the program’s impact on postpregnancy FP uptake during extended postpartum and postabortion periods; the feasibility and acceptability of introducing programmatic elements and new methods of postpregnancy FP; and the potential for scaling up successful programmatic elements and interventions ( Table 1 for study themes and key questions). Collectively, these questions aim to assess whether or not PPFP Choices has successfully led to the Outcomes listed in the Theory of Change ( Figure 1). The expectation is that if the outcomes are fully met and recommendations for scale-up of similar programs and sustainability of quality FP services are accepted, then, over time, impacts will also be met.

**Table 1.** PPFP Choices study themes and key questions to be explored.

Study theme/question
What are the key determinants at service delivery, provider, and client levels that influence the uptake of postpregnancy family planning in the public and private health care sectors in Indonesia and Kenya?
**Programmatic effort** • What programmatic inputs increase a woman’s likelihood of accepting a FP method immediately postpartum or postabortion? • What are the costs associated with implementing such interventions? • What are the costs and programmatic efforts needed to scale up and sustain these interventions? • What are the barriers and facilitators for young women’s accessing postpregnancy FP services? • What are effective programmatic approaches to engaging the private sector to provide a full range of postpregnancy (FP) methods?
**Feasibility** • What proportion of postpregnancy women receive an FP method, according to standards, prior to discharge, among those who opted for an FP method, by age, type of client, timing, and method? • To what extent was the health care system able to offer a full range of postpregnancy FP methods within existing service delivery platforms? (Areas to be examined will include infection prevention, resources, practices, knowledge and skills, commodity supply chain management, labor and delivery staffing, workflow, etc.) • To what extent were the health care facilities offering appropriate and quality postpregnancy FP counseling at all relevant time points (antenatal care, early labor, prior to discharge, follow-up visits)? • To what extent were providers able to provide, with technical quality, FP counseling and services within the immediate postpregnancy period? • What are internal and external inputs that incentivize or disincentivize private sector facilities in the provision of postpregnancy FP services?
**Acceptability** • What proportion of women postpregnancy choose a FP method after FP counseling? What proportion receive the method in the immediate postpregnancy period prior to pre-discharge? • What proportion of women received a different FP method in the immediate postpregnancy period than the one they opted for prior to delivery or uterine evacuation? What were the determining factors in this difference? • To what extent do providers and women understand the benefits of FP in the immediate postpregnancy period? • To what extent do providers accept the need to provide postpregnancy FP counseling and service provision? • What was the continuation rate (within six months postpregnancy) and reasons for discontinuation for those no longer using the method? • What proportion of women using the lactational amenorrhea method transition to another contraceptive method by six months postpartum? To what methods? • At six months postpartum, are there differences in FP uptake between women exposed to the PPFP Choices interventions compared with women in the control settings? • Do women exposed to the PPFP Choices interventions exhibit different contraceptive use behaviors (uptake, discontinuation, switching) in their first year postpartum, compared with women in control settings? (In Kenya only)
**Safety** • What were the rates of minor, moderate, and major adverse events? • Were women experiencing adverse events treated to standards?
**Scalability** • When scaling up postpregnancy interventions, who are the key players and what are the key factors? What works and what doesn’t? Are there differences between public and private sector? • What contributes to the success of implementing and scaling up of postpregnancy FP in the private sector? • When and how do women interact with health care providers regarding postpartum FP in the first year postpartum? (In Kenya only)

PPFP Choices, Post Pregnancy Family Planning Choices; FP, family planning.

## Methods

### Study design

The PPFP Choices study is a multi-country, quasi-experimental operations research study with intervention and control groups, implemented in collaboration with *Kementerian Kesehatan Republik Indonesia* (KemKes, the Indonesian Ministry of Health) and the Kenyan MOH. In Indonesia, the study is implemented in the Brebes District as the intervention area and the Batang District as the control area, both in the Central Java Region. In Kenya, the PPFP Choices study team chose Meru County as the intervention area with Kilifi County as the control area. Selection criteria are described in the *Study setting* section.

A mixed methods approach is being used for study activities; both quantitative and qualitative data is collected through interviews and assessments longitudinally at multiple time points. Study participants are ANC, postpartum, and postabortion clients at study facilities. Quantitative interviews are completed with study participants at ANC (in Kenya only), immediately postpartum or postabortion, six months following delivery or postabortion care, and 12 months following delivery (Kenya only). Focus group discussions (FGDs) and in-depth interviews (IDIs) take place with subsets of study participants between six and 12 months postpartum and postabortion. Data collection from individuals receiving care at study facilities will aid in answering questions related to the efficacy, feasibility (including safety) and acceptability of the intervention at facility level. Key informant interviews (KIIs) are undertaken at baseline and endline with community influencers and facility providers and managers. Facility assessments of each study facility take place at baseline, midline, and endline, while facility service statistics are gathered monthly. These discussions aim to determine the acceptability of the intervention at subnational and national levels and identify the program efforts required scale-up to intervention for broader use across the country. Given that components of PPFP Choices’ package of interventions are implemented in an as-needed manner, there is also an intervention tracker tool, which the study team created to keep track of intervention activities taking place.

### Sample sizes

Separate sample sizes were calculated for Indonesia and Kenya and pregnant/postpartum and postabortion women cohorts. Once the sample size for pregnant/postpartum women was determined, it was then used to identify the number of facilities needed in each country.

For pregnant/postpartum women, our estimates aim to measure a difference in the six-month postpartum acceptance of long-acting reversible and permanent methods by postpartum women seeking services at control and intervention facilities. Sample size calculations for both countries are based on a 95% two-sided confidence interval with 80% power. The sample size was calculated in two stages. First, we calculated the sample size for simple random sampling. Next, the sample size was adjusted to take into account a design effect of 2.5 to adjust for within-cluster correlation and non-response.

In Indonesia, we used a re-analysis of FP utilization from the Indonesia 2012 Demographic and Health Survey (DHS) (referencing 10.9% of married women in Indonesia were using a LARC or PM at six-months postpartum) and conversations with the MyChoice project team to estimate a baseline LARC+PM use of approximately 10% at six months postpartum by clients experiencing usual ANC, labor and delivery and postpartum care. In Kenya, we analyzed FP method use among a subset of 3,857 women with a child under one year who were interviewed during the 2014 Kenya DHS to estimate a LARC+PM use rate of 6% at six months postpartum by clients experiencing usual ANC, labor and delivery and postpartum care. From discussion with key stakeholders, we estimated that PPFP Choices would lead to an increase of 50% in Indonesia, where MyChoice was already operating, to 15% of women using a LARC+PM method at six months postpartum. In Kenya, where no existing program was in place, we estimated the percent of women using a LARC+PM method at six months postpartum would increase by two-thirds, to 10%. Sample sizes were therefore calculated to measure a difference in use of a LARC+PM method at six months postpartum of 10% of women recruited at comparison facilities compared to 15% of women recruited at intervention facilities in Indonesia. In Kenya, sample sizes aim to estimate a difference of 6% of women recruited at comparison facilities and 10% of women recruited at intervention facilities over the course of the study. We have further estimated a need for at least 20% of the sample to be recruited from private facilities to effectively measure changes in both types of facilities, resulting in sample sizes of 4,288 and 4,508 women in Indonesia and Kenya, respectively.

For postabortion women, since the current proportion of these women who take up an FP method after receiving postabortion care has not been reliably determined in the two countries, we have assumed an estimate of 50% (assumption of 50% provides us a high sample size). After six months, we estimate that 40% of them will still be using the method, and thus we need a sample size of 243 women in the intervention group only, per country. This group was recruited at baseline, and included an adjustment of 20% to account for loss to follow-up. This sample size is adequate to detect a net 10 percentage point change within a 95% two-sided confidence interval at 80% power.

To reach the sample sizes of 4,288 and 4,508 women in Indonesia and Kenya, respectively, we then determined that, based on the client volumes of eligible health facilities, in Indonesia, three public and one private health facilities per arm (eight total) were needed, and in Kenya, there five public and six private facilities per arm (22 total) were needed for a one year recruitment period. The breakdown of public and private health facilities is intended to capture the differences between the two in each country. The selection of the health facilities is based on the matching process described in the *Study setting* section. We anticipate these eight facilities in Indonesia and 22 facilities in Kenya will also be sufficient to reach the sample size needed for postabortion clients during the recruitment period.

A subset of the women recruited for structured interviews will be asked to participate in in-depth interviews (IDIs) or FGDs. In Kenya, we expect to interview 32 postpartum adult public facility participants in four groups of eight FGD participants per study arm, 16 postpartum adult private facility participants per arm via IDIs and 16 postpartum public and private adolescent participants per arm via IDIs. In Indonesia, we expect to interview 64 postpartum adult public and private facility participants in eight FGD groups (4 public facility FGDs and 4 private facility FGDs) per study arm and 16 postpartum public and private adolescent participants per arm via IDIs. In both countries, we expect to interview 8 public or private facility postabortion participants via IDI.

### Study setting

In Indonesia, where the MyChoice project has been strengthening postpartum FP since 2015, we chose the Brebes and Batang Districts in consultation and discussion with MOH and Jhpiego colleagues based on the larger project implementation and scale-up plans in the coming years. The selection was based on size and characteristics of the potential study population including: number of public and private facilities, number of facility ANC visits per year, proportion of women attending four or more ANC visits, number of deliveries per year, and programmatic naivety based on our knowledge that there were no other ongoing or anticipated relevant interventions that could potentially introduce bias. Districts were matched as much as possible across the selection criteria. While both districts are in Central Java, they are not contiguous and there is little chance of contamination between districts. We chose Brebes to be the intervention district as, in Indonesia, the PPFP Choices Intervention was to build upon the ongoing MyChoice intervention at the time. Brebes received the MyChoice and PPFP Choices intervention shortly before PPFP Choices study data collection while Batang was scheduled to receive the intervention in coordination with the MyChoice project scale-up after the PPFP Choices study data collection is completed. In Kenya, counties eligible to be study areas met the following criteria: as reported by the Kenya 2014 DHS; 30,000 or more women are seen per year for their first ANC visits (higher than the Kenya country median of 21,881); the number of normal deliveries in the county was above the median of 12,775; the proportion of women attending four or more ANC visits is above the Kenya median of 56%; more than the country median of 54% of women deliver in the hospital; the number of deliveries was equal or more than the ANC clients; and to the best knowledge of the PPFP Choices study team, no other similar FP programs were planned for the next three years. Upon comparing counties with these criteria, we chose Meru and Kilifi counties and randomly chose Meru to be the intervention county and Kilifi to be the control county. Similar to Indonesia, counties were matched as much as possible across the selection criteria and as they are not contiguous, there is little expectation of contamination from intervention area to control area.

Health facilities within the selected study county/district were determined based on meeting the following criteria, as determined during facility assessments undertaken by PPFP Choices staff:

- The health facility has provider(s) trained and/or who can be trained to provide relevant postpregnancy FP counseling and services
- The distance/location and accessibility of the health facility is programmatically feasible for introducing study interventions
- The health facility is within or serves the specific study county/district
- The health facility is legally registered and current in its registration with the host-country government
- The health facility is either public or private for-profit (indigenous owned, tax paying)

We then matched facilities across the intervention and control areas by: ownership type (public or private), number of new ANC visits, number of normal deliveries and number of postabortion care cases (as a proxy for postabortion care services provided). Based on the sample sizes needed, we then matched three private and one public facilities per arm (a total of eight study facilities) in Indonesia and five private and six public facilities per arm (a total of 22 study facilities) in Kenya.

### Type of participants and process for recruitment and consent

After the health facilities were identified, we liaised with the local ministries of health and individual health facility leadership to obtain permission to conduct the study at the selected facilities.

There are three distinct types of participants: 1) pregnant or postpartum clients who participate in client interviews and FGDs or IDIs; 2) postabortion clients who participate in client interviews and in-depth interviews; and 3) policy, facility, or community level leaders who are engaged through key informant interviews.

*Pregnant or postpartum participants:* Pregnant or postpartum participants in Indonesia are engaged in quantitative interviews at two separate time points—at discharge after delivery or postabortion care and at six months postpartum or postabortion. In Kenya, they are engaged in quantitative interviews at four separate time points—at ANC, at discharge following delivery or postabortion care, six months following delivery or postabortion care, and 12 months following delivery. The first interview for Kenyan ANC/postpartum participants takes place at an ANC visit. The differences were based on an initial assessment in preparation for the study in April 2016; the study team determined that while the majority of Kenyan ANC clients attend the same facility for ANC visits and labor and delivery (L&D), Indonesian clients are less likely to do so. To reduce higher than optimal loss to follow-up between an ANC and L&D, the study team decided to recruit postpartum women in Indonesia when they attend L&D care instead of at ANC. In Kenya, in consultation with study donors, the fourth interview at 12 months postpartum was added to the study protocol after data collection had begun but before the majority of the participants in Kenya had passed 12 months postpartum. Unfortunately, a majority of women in Indonesia had already passed the 12 months postpartum time point when the 12 months interview was designed and we were unable to add this component to the study plan in Indoensia. A subsample of all postpartum participants are also interviewed through FGDs or IDIs.

Pregnant/postpartum women who are eligible for participation in the PPFP Choices study met the following study criteria:

- Indonesia specific:
  a. In the immediate postpartum period (within 72 hours, prior to leaving the health facility)
  b. Reports having attended ANC within her third trimester (28 weeks pregnant and later) at a study facility

- Kenya specific:
  a. At least 28 weeks pregnant
  b. Reports that she plans to deliver at a study facility

- Both Indonesia and Kenya:
  a. Aged 15–49 years at enrollment (Indonesian adolescents aged 15–16 must be married for purposes of the study consent. Indonesian adolescents who are married, as well as Indonesian adolescents who are 17 years old and older are considered legal adults. Pregnant adolescents in Kenya are considered legal adults.)
  b. Provides voluntary informed consent
  c. Does not plan to relocate in the next 12 months at the time of enrollment

*Postabortion participants:* In both countries, postabortion participants are engaged in quantitative interviews at two separate time points, and a subsample are also interviewed through in-depth interviews following their second quantitative interview. Postabortion women who are eligible for participation in the PPFP Choices study met the following study criteria:

- In the immediate postabortion care period (within 72 hours in Indonesia, within 48 hours in Kenya, prior to leaving the health facility for treatment of an incomplete abortion)
- Aged 15–49 years at time of enrollment (Indonesian adolescents aged 15–16 must be married for purposes of the study consent. Indonesian adolescents who are married, as well as Indonesian adolescents who are 17 years old and older are considered legal adults. Pregnant adolescents in Kenya are considered legal adults.)
- Provides voluntary informed consent
- Does not plan to relocate in the next six months

Recruitment for all pregnant, postpartum, and postabortion participants is completed at the study facilities with standardized recruitment, screening, and consent tools. ****

*Key informant participants*: In both countries, key informant participants represent key groups of interest who understand the individual, community, and institutional factors affecting postpregnancy FP within their respective country and region. They include representatives from the MOH and other policy makers, religious and community influencers, public and private facility health care providers, and health facility administrators. Key informants are interviewed at the beginning of the PPFP Choices project and after completion of all client participant enrollment (prior to implementation of the PPFP Choices package of interventions in the control areas). Key informants who are eligible for participation in the PPFP Choices study:

- Currently live or work in a study region
- Are at least 18 years old
- Understand the local language (Bahasa in Indonesia and Kiswahili or Kimeru in Kenya) or English
- Hold an authoritative, political, or programmatic position that could influence issues affecting access to postpregnancy FP
- Able to provide voluntary informed consent
- Agree to the audio recording of the discussion

Key informants are purposely selected by the study team on the basis of their potential to influence postpregnancy FP, familiarity with the culture and community, and ability to communicate. They are recruited, consented, and interviewed by higher-level study staff using standardized tools. KIIs are done at the initiation of the study in part to inform the intervention, and again at the end of the study to assess if knowledge and understanding of these key groups changed.

Written informed consent will be sought from all participants before any data collection begins. Materials will be provided to participants in English, Bahasa, Kimeru or Kiswahili and consent will be obtained confirmed on signed informed consent forms bearing either the participant’s initials, signature or a thumbprint. A signed copy of the consent form was given to the participant for their retention. In case a participant did not wish to take the copy, both signed copies would be kept in the study folder.

### Study instruments

**Figure 3.**
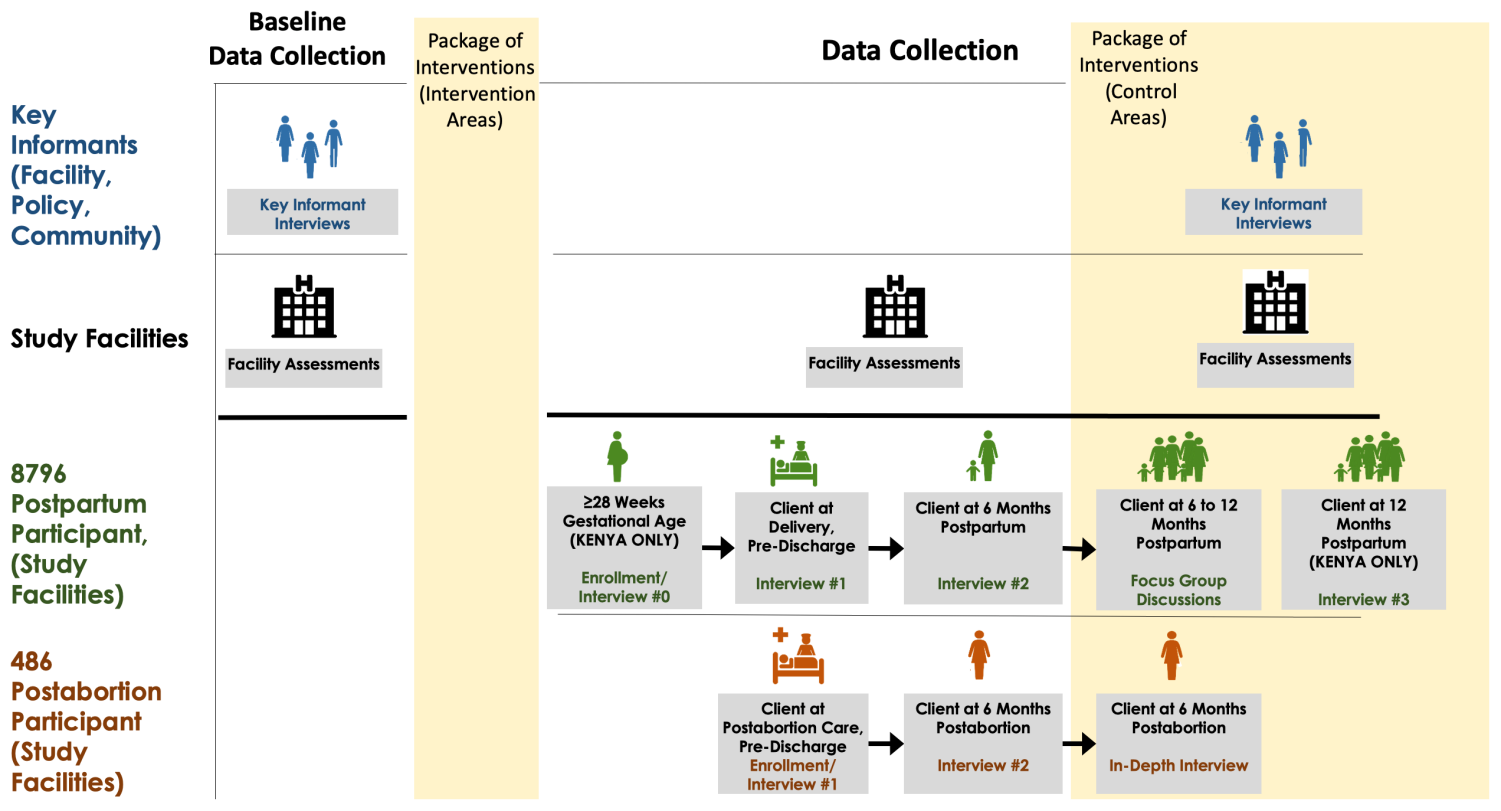
Post Pregnancy Family Planning Choices data collection method by participant type and time point.

Following initial implementation of the packages of interventions, the PPFP Choices data is collected through a mixed methods approach. Quantitative data is collected about each facility using facility assessments and data extraction from facility records, and from postpartum and postabortion participants using client interviews. Qualitative data, used to complement and further develop themes uncovered in the quantitative data, is gathered from a subset of postpartum and postabortion participants through FGDs and IDIs, and from purposely selected from interviews with key informants. All data collection tools were created with technical expert input and underwent multiple iterations and reviews through rounds of in-country pretesting prior to the start of data collection. In Indonesia, pre-testing of data collection tools was completed in Central Java with support from the Center for Health Policy and Management at the University of Gadjah Mada, whom we contracted for data collection. In Kenya, PPFP Choices staff pre-tested each tool with help from participants at Nairobi-based health facilities. Each study tool has specific goals and is administered at the different time point of the study as described below. All English, Kiswahili, Kimeru, and Bahasa versions of the study tools are accessible on Figshare in the project Post Pregnancy Family Planning Choices in the Public and Private Sectors in Kenya and Indonesia ^17– 36^. Figure 3 summarizes types of data collection method by participant and time point.

*Facility assessment:* Facility assessments take place at three points throughout the study period: baseline, midline, and endline. The assessment gathers information on staff cadre, numbers in each cadre, and reported service provision ability; average monthly numbers of selected pregnancy and FP-related services provided; and select FP-related commodity and equipment availability. In addition, data collectors extract monthly service provision statistics from registers for each health facility. Data recorded include facility-wide numbers of monthly ANC, delivery, and postabortion care clients as well as reported FP counseling and method provision for those clients.

*Postpartum client interviews:* The goal of the ANC, postpartum, and postabortion client interviews is to understand the relationships between client experiences, satisfaction, and attitudes; postpregnancy FP knowledge and FP intentions; and use and continuation of FP. Face-to-face interviews take place between the client and a data collector at each collection point, with the data collector recording all answers directly.

In Indonesia, postpartum clients are first approached for recruitment by PPFP Choices data collectors during their delivery visit, immediately prior to discharge from the health facility, after all other visit activities have been completed. Eligible women who consent to participation are interviewed immediately following recruitment. This interview is known as Interview #1 and will gather information on experiences and FP intentions both at ANC (retrospectively) and in the immediate postpartum period. Interview #2 will take place when the client is between six and seven months postpartum and will collect information on FP-related use, intentions, and knowledge within the first six months postpartum. For this interview, data collectors will have, with prior permission, called or visited the participant to set the interview time and place.

In Kenya, pregnant and postpartum clients are first approached for recruitment at an ANC visit at 28 weeks gestation or later. They are approached by the data collectors immediately after all other visit activities are completed. Eligible women who consent to participation are interviewed immediately following recruitment at the ANC visit. This is known as Interview #0. As with the Indonesian participants, Interview #1 will take place in the immediate postpartum period (within 48 hours after delivery) after completing the delivery visit but prior to leaving the health facility. Subsequently, if an expected participant has not been found at the facility by the data collector two weeks after her estimated delivery date, the data collector will contact the participant by mobile phone or a home visit to complete a No-Show Follow-Up Interview. The No-Show Follow-Up Interview will take the place of Interview #1 and will collect participant demographics and delivery location. As with Indonesian participants, Interview #2 will take place when the client is between six and seven months postpartum and will collect information on FP-related use, intentions, and knowledge within the first six months postpartum. In Kenya an additional Interview #3 will take place when the participant is 12–18 months postpartum and are assessed on their full first-year postpartum participant experiences with FP and health facility visits. For interviews #2 and #3, data collectors will have, with prior permission, called or visited the participant to set the interview time and place.

Qualitative interviews will take place with a subset of postpartum participants between six and 12 months postpartum with the goal of expanding upon learnings from the quantitative interviews to further understand relationships between client satisfaction and attitudes, postpregnancy FP knowledge and FP intentions, and use and continuation. Potential participants are randomized and invited by study researchers to participate in the FGDs (at a set time and location) and IDIs (at a time and location agreed upon by the researcher and participant).

*Postabortion client interviews:* In both countries, postabortion participants are recruited by data collectors in the immediate period following receipt of postabortion care (within 48 hours in Kenya and within 72 hours in Indonesia) after all visit activities are complete and prior to discharge from the facility. All eligible women who consent to participation are interviewed for Interview #1 immediately following recruitment. As with postpartum participants, Interview #2 will take place between six and seven months postpartum after the data collector, with prior permission, called or visited the participant to set the interview time. IDIs will take place with a subset of postabortion participants immediately following their interview #2. As with the postpartum FGDs and IDIs, the postabortion IDIs are invited at random prior to the interview time point.

*KIIs:* KIIs are completed at two time points; study baseline and endline. Interviews take place with individuals purposefully selected to represent key groups of interest that understand the individual, community, and institutional factors affecting postpregnancy FP within the countries as a whole or the specific study areas. Key Informants are invited to be interviewed, consented, and interviewed by Jhpiego PPFP Choices study leaders from the country offices.

The numbers of FGDs and IDIs in Indonesia and Kenya were determined by the number of overall participants available in each interview category. See Table 2 for data collection method and its goal by sample size of each participant type and time point.

**Table 2.** Data collection method by participant type, goal, and time point.

Data collection method	Number and description of sample	Goal	Time point
**Key informant** **interviews** **(KIIs)**	**In each country**: 1 national ministry of health official 2 county/district level ministry of health and policy makers 2–3 community influencers 3 public facility providers and administrators 3 private facility providers and administrators	Identify social, cultural, contextual, economic, and age- and gender-related factors impacting community perceptions of and barriers to accessing and utilizing FP services	Baseline and endline
**Client** **interviews**	**In Indonesia**: 4,288 postpartum women and 243 postabortion women who gave birth or accessed postabortion services at a study facility	Understand relationships between client satisfaction and attitudes, PPFP knowledge and FP intentions, and use and continuation	Antenatal care (Kenya only), immediate postpartum/postabortion, 6 months postpartum/ postabortion, and 12 months postpartum (Kenya only)
	**In Kenya**: 4,508 postpartum women and 243 postabortion women who gave birth or accessed postabortion services at a study facility		
**Focus group** ** discussions**	**In Indonesia**: Two groups of 8 adult public facility FP acceptors per arm Two groups of 8 adult private facility FP acceptors per arm Two groups of 8 public non-acceptors per arm Two groups of 8 private non-acceptors per arm Total = 128 adult women 6–12 months postpartum	Identify social, cultural, contextual, economic, and age- and gender-related factors affecting women’s perceptions of, access to and use of FP	6–12 months postpartum
	**In Kenya**: Two groups of 8 adult public facility FP acceptors per arm Two groups of 8 public non-acceptors per arm Total = 64 adult women in public facilities 6–12 months postpartum		
**In-depth** **interviews**	**Postpartum adolescents in each country**: 8 adolescent public facility participants per arm 8 adolescent private facility participants per arm Total = 32 adolescent women 6–12 months postpartum	Identify social, cultural, contextual, economic, and age- and gender-related factors affecting adolescent women’s perceptions of, access to and use of FP	6–12 months postpartum
	**Kenya postpartum private facility adults**: 8 adult private facility FP acceptors per arm 8 adult private facility non-acceptors per arm Total = 32 adult women in private facilities 6–12 months postpartum		
	**Postabortion women in each country**: 6–12 months postpartum Total = 8 women from public or private facilities per country	Identify social, cultural, contextual, economic and age- and gender-related reasons women did not receive an immediate postabortion FP method	6–12 months postabortion
**Facility** **assessments**	**In Indonesia**: 4 study facilities per arm (8 total)	Identify facility-level supply-side barriers to accessing and utilizing PPFP	Baseline, midline, and endline
	**In Kenya**: 11 study facilities (22 total)		

PPFP Choices, Post Pregnancy Family Planning Choices; FP, family planning.

### Data entry, analysis and quality assurance

All quantitative study data is collected and managed through the use of REDCap (Research Electronic Data Capture) ^37^, a secure web-based electronic data collection platform. The Jhpiego REDCap server is hosted in Jhpiego Kenya’s Nairobi office. All qualitative study data is collected via audio recordings that are transcribed to Microsoft Word then analyzed using ATLAS.ti, a qualitative data analysis and research software.

Upon data capture, all quantitative data undergoes cleaning and quality assurance processes prior to analysis. The PPFP Choices REDCap system is equipped with validation, range, and consistency checks to minimize data entry errors. Immediately following initial data capture, and progressively throughout the study, data reviews are completed by a program manager and data manager, further minimizing any data collection errors. All electronic data entry systems are password protected for individual users.

After all quantitative datasets are thoroughly cleaned, the study team will report on study outcomes. Mixed-effects regression models will be used to explore a primary outcome measuring the differences in acceptance of postpartum FP six months after delivery between the intervention and control facilities, different demographic groups and to compare acceptance among clients receiving varying levels of care. Models will initially include a random intercept for facility, but may also explore the effects of facility-level variables (for example, public versus private facilities) on outcomes. As women will receive care from multiple providers over the course of the study, we do not intend to include provider-level variables. However, we intend to develop composite variables to measure the effect of quality of care and FP counseling on postpartum use. Survival analysis techniques may also be used to explore time-to-method acceptance for postpartum clients.

Qualitative datasets will be analyzed using grounded theory methods. Initial interviews, transcribed and translated into English, will be coded and analyzed in ATLAS.ti using both *a priori* codes and themes that emerge during the coding process. All datasets prepared for quantitative and qualitative analysis will have been be de-identified and will be made available to the public per agreement with donors.

### Ethical considerations

PPFP Choices is implemented with institutional review board approval from the Kenya Medical Research Institute (KEMRI, Protocol number non-KEMRI 521), the Indonesian MOH (KemKes, number LB.02.01/5.2/KR.002/2017), and the Johns Hopkins Bloomberg School of Public Health (JHSPH, IRB number 00007462). The study team strictly follows a study manual with a series of standard operating procedures (SOPs) capturing all potential events to the extent possible. Included in the SOPs are instructions for actions to be taken and documentation criteria if any deviations or unanticipated events take place.

While the study team does not anticipate that any adverse events will occur as a result of participation in the study, the team does anticipate that infant and maternal deaths, not related to the study interventions, will take place among the study population. Based on each country’s most recent (at the time of study commencement) infant mortality rate and maternal mortality rate available at the time of study inception (2012 Indonesia Demographic Health Survey ^38^ and 2014 Kenya Demographic Health Survey ^39^), we expect the study will encounter 137 infant deaths and 15 maternal deaths in Indonesia and 176 infant deaths and 16 maternal deaths in Kenya. Per PPFP Choices’ study manual and SOP, upon encounter of any expected or unexpected adverse events, the data collectors are to immediately report to the study team. The study team will carefully review each incident and report to JHSPH, KEMRI, and KemKes when appropriate.

### Study status

**Figure 4.**
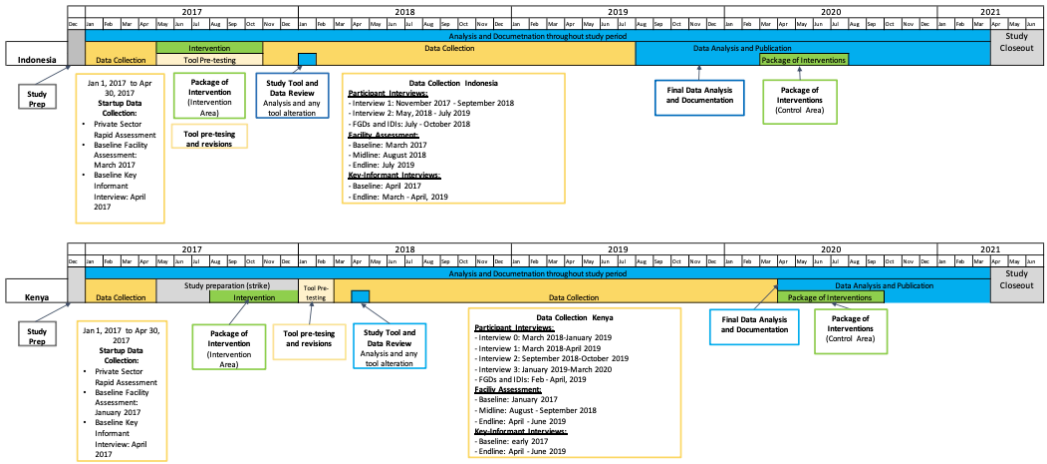
PPFP Choices study timeline.

The PPFP Choices study completed recruitment in January 2019 and ended all follow-up data collection March 2020, a few weeks prior to the anticipated end of data collection due to threat of pandemic., a few weeks prior to anticipated end date due to the threat of pandemic. As of June 2020, data verification and cleaning has been completed and the study team is currently analyzing all study data. A detailed study timeline is displayed in Figure 4 below.

## Discussion

By the end of PPFP Choices, we hope to generate and disseminate actionable evidence of positive drivers, barriers, and activities that do not yield results with regard to increasing uptake of postpregnancy FP and institutionalizing postpregnancy FP in the public and private sectors in Indonesia and Kenya. More importantly, these learnings and experiences will contribute to the global efforts to advance and scale up postpartum and postabortion FP in similar settings beyond these two countries.

We anticipate the study’s data collection will be completed by April 2020 and we will begin dissemination of the most important and relevant program learnings beginning in mid-2020. In Indonesia, the government has already committed to reach 80% of postpartum women with FP services but more needs to be done to incorporate and include more integrated postpregnancy FP services. In Kenya, we expect the results will encourage the government and stakeholders to embrace a more comprehensive postpregnancy FP scale-up plan. Globally, countries are moving toward universal health coverage ^40^ and more women will be giving birth in facilities, presenting an enormous opportunity to provide postpartum FP to those who want it. Postabortion FP can also help women and girls achieve their reproductive intentions and provide cost savings for both clients and the health system ^8^. Instead of introducing stand-alone interventions on postpartum FP or postabortion FP, a comprehensive postpregnancy FP package of interventions can hopefully be implemented with the actionable evidence from PPFP Choices. Dissemination of PPFP Choices’ program reports, lessons learned, and research findings will target multi-sectoral stakeholders, including the global FP community, country-level policy makers, national and subnational governments, implementing partners, and local non-profit organizations via a variety of fora.

Aside from actionable evidence generated around postpregnancy FP programming, it is also expected that this multi-country study will provide valuable lessons learned from a study methodology point of view. The lessons might include ways to minimize loss to follow-up with postpregnancy women during this vulnerable period of time in these settings. Additionally, there will also be lessons around data management processes for two countries with similar but not identical study questionnaires, topics may include analysis of quasi-experimental operations research data collected at different time points from study cohorts who may or may not be exposed to the exact same intervention.

## Data availability

### Underlying data

No underlying data are associated with this article.

### Extended data

Figshare: PPFP Choices Kenya Postpartum Interview 0. https://doi.org/10.6084/m9.figshare.12475760.v1 ^17^

Figshare: PPFP Choices Kenya Postpartum Interview 1. https://doi.org/10.6084/m9.figshare.12485360.v1 ^18^

Figshare: PPFP Choices Kenya Postpartum Interview 2. https://doi.org/10.6084/m9.figshare.12485375.v1 ^19^

Figshare: PPFP Choices Kenya Postpartum No-Show Follow-Up Interview. https://doi.org/10.6084/m9.figshare.12485387.v1 ^20^

Figshare: PPFP Choices Kenya Postpartum Interview 3. https://doi.org/10.6084/m9.figshare.12485429.v1 ^21^

Figshare: PPFP Choices Indonesia Postpartum Interview 1. https://doi.org/10.6084/m9.figshare.12485996.v1 ^22^

Figshare: PPFP Choices Indonesia Postpartum Interview 2. https://doi.org/10.6084/m9.figshare.12486011.v1 ^23^

Figshare: PPFP Choices Kenya and Indonesia Postabortion Care Interview 1. https://doi.org/10.6084/m9.figshare.12485771.v1 ^24^

Figshare: PPFP Choices Kenya and Indonesia Postabortion Care Interview 2. https://doi.org/10.6084/m9.figshare.12485801.v1 ^25^

Figshare: PPFP Choices Kenya and Indonesia Adult PPFP Acceptor Focus Group Discussion Guide. https://doi.org/10.6084/m9.figshare.12494663.v1 ^26^

Figshare: PPFP Choices Kenya and Indonesia Adult PPFP Non-Acceptor Focus Group Discussion Guide. https://doi.org/10.6084/m9.figshare.12494669.v1 ^27^

Figshare: PPFP Choices Kenya Adult Private Facility PPFP Acceptor In-Depth Interview Guide. https://doi.org/10.6084/m9.figshare.12494501.v1 ^28^

Figshare: PPFP Choices Kenya Adult Private Facility PPFP Non-Acceptor In-Depth Interview Guide. https://doi.org/10.6084/m9.figshare.12494582.v1 ^29^

Figshare: PPFP Choices Kenya Adolescent Postpartum In-Depth Interview Guide. https://doi.org/10.6084/m9.figshare.12494375.v1 ^30^

Figshare: PPFP Choices Indonesia Adolescent Postpartum In Depth Interview Guide. https://doi.org/10.6084/m9.figshare.12494660.v1 ^31^

Figshare: PPFP Choices Kenya and Indonesia Postabortion In-Depth Interview Guide. https://doi.org/10.6084/m9.figshare.12494600.v1 ^32^

Figshare: PPFP Choices Kenya and Indonesia Community Influencer Key Informant Interview Guide. https://doi.org/10.6084/m9.figshare.12494615.v1 ^33^

Figshare: PPFP Choices Kenya and Indonesia Facility Administrator and Providers Key Informant Interview Guide. https://doi.org/10.6084/m9.figshare.12494648.v1 ^34^

Figshare: PPFP Choices Kenya and Indonesia Policy Maker Key Informant Interview Guide. https://doi.org/10.6084/m9.figshare.12510698.v1 ^35^

Figshare: PPFP Choices Kenya and Indonesia Facility Assessment Tools. https://doi.org/10.6084/m9.figshare.12541559.v1 ^36^

Figshare: PPFP Choices Kenya and Indonesia List of Activities Implemented. https://doi.org/10.6084/m9.figshare.13318742.v1 ^16^

Data are available under the terms of the Creative Commons Zero "No rights reserved" data waiver (CC0 1.0 Public domain dedication).
