# Supplementary material for: Study protocol for Post Pregnancy Family Planning Choices, an operations research study examining the effectiveness of interventions in the public and private sectors in Indonesia and Kenya
Source: Gates Open Res. 2020 Dec 18;4:89. Originally published 2020 Jul 2. [Version 2] doi: 10.12688/gatesopenres.13147.2 (PMC7919138; doi:10.12688/gatesopenres.13147.2)
Supplement: Peer review history

# Peer review: Reviewer response for version 2

**DOI:** 10.21956/gatesopenres.14425.r30146

**Published:** 2021-02-26

**Authors:** Saumya Ramarao, Avishek Hazra

**Affiliations:** 1 Population Council, New Delih, Delhi, India; 2 Population Council, New York City, NY, USA

I approve of the revisions made by the authors.

Is the study design appropriate for the research question?

Yes

Is the rationale for, and objectives of, the study clearly described?

Yes

Are sufficient details of the methods provided to allow replication by others?

Partly

Are the datasets clearly presented in a useable and accessible format?

Not applicable

Reviewer Expertise:

NA

We confirm that we have read this submission and believe that we have an appropriate level of expertise to confirm that it is of an acceptable scientific standard.

# Peer review: Reviewer response for version 2

**DOI:** 10.21956/gatesopenres.14425.r30147

**Published:** 2021-01-06

**Authors:** John Cleland

**Affiliations:** 1 Department of Population Health, London School of Hygiene & Tropical Medicine, London, UK

Thank you for your detailed responses. I have no further suggestions. I do note however that lack of cost data remains a weakness.

Is the study design appropriate for the research question?

Partly

Is the rationale for, and objectives of, the study clearly described?

Yes

Are sufficient details of the methods provided to allow replication by others?

No

Are the datasets clearly presented in a useable and accessible format?

Not applicable

Reviewer Expertise:

Demography, fertility, family planning

I confirm that I have read this submission and believe that I have an appropriate level of expertise to confirm that it is of an acceptable scientific standard.

# Peer review: Reviewer response for version 1

**DOI:** 10.21956/gatesopenres.14338.r29035

**Published:** 2020-09-09

**Authors:** Saumya Ramarao, Avishek Hazra

**Affiliations:** 1 Population Council, New York City, NY, USA; 2 Population Council, New Delih, Delhi, India

**1) Information does exist in Kenya regarding postabortion family planning uptake. See for example:**

1. Mackenzius *et al.* (2018 ^1^).
2. Wendot *et al.* (2018 ^2^).

**2) Abstract:**

1. In the Method section it’s written ‘Participants include health facilities.” Since facilities are not participants as such, please indicate if by participant the intention was to refer to health personnel. 
2. The method section should also include i) the duration of the operations research, ii) the targeted sample sizes for quantitative and qualitative interviews (a break up by public and private may also be useful), and iii) the time points (year) of each wave of data collection.

**3) Introduction:**

1. This is well written. ‘The study follows a theory of change model’. You may like to specify if the study team developed this ToC or adopted/adapted from other existing ToC. The ToC is too crowded and wondering if a simplified version with key phrases could be used instead of long sentences in the ‘inputs’, ’process’….‘impact’ blocks.
2. It’s also not very clear if the study will assess all the outcomes and also impacts. It’s worth specifying which components of the ToC the study will measure/assess.

**4) Methods:**

1. Were the experimental and control districts matched? Are they contiguous? Is there any chance of contamination between the intervention and control districts?
2. It was not very clear if baseline data had already been collected. It seems that interventions started in 2017 suggesting that the baseline would have had to happen prior to 2017. Please clarify.
3. It will be helpful if the authors could provide a diagram to describe the design as is typical of OR studies - (i.e.) the diagram should include the time points of data collection (t1,t2), the experimental and control arms (E/C), and the intervention (x).
4. Glad to see that the sample size calculations accounted for clustering and the design effect.
5. Please clarify if “Sample sizes were calculated to measure a change from 10% to 15% in Indonesia and from 6% to 10% in Kenya at intervention facilities over the course of the study.” Please state clearly if these are the ‘absolute’ changes in the intervention area OR, these are ‘net’ changes in the intervention area accounting for the change in the control area?
6. More importantly, the prevalence rates considered for sample size calculation are of 2012 and 2014, and until the time the project began, there could be changes in these rates. Did your sample size calculation consider/factored that in and if so please specify how.  Similarly, for post-abortion women, the assumed 10% points change is ‘absolute’ or ‘net’ change?
7. Please specify the target sample size for the qualitative interviews.
8. Please specify the duration of the study so that it is easier for a reader to know over what period the hypothesized change would occur.  “change from 10% to 15% in Indonesia and from 6% to 10% in Kenya at intervention facilities over the course of the study.”
9. A strength of the proposed design is the longitudinal tracking of women.
10. Include some information on what types of cost data will be collected.
11. State clearly whether the respondents in the ‘multiple time points’ are cross-sectional or longitudinal cohort of women in the study design section (the mention of cohort appears much later). Also, please specify the ‘multiple’ time points in terms of year/months of surveys.
12. Was the voluntary informed consent verbal or written? In which language? Was a copy of the consent form given to them?

**5) Analysis:**

1. Please add a couple of lines to indicate that the analytical approach will be a difference in difference method. This seems to be implicit but make it explicit. If so, please state clearly what would be the dependent variables and independent variables that would be adjusted for.
2. Indicate the different levels that will be considered in the multilevel analysis.
3. It will also be helpful to include explicitly how public and private facilities will be compared since private sector involvement is a key component of the study.
4. In addition to facility level variation, there will likely be provider level variations that will need to be taken care of. Unless in certain types of facilities, there is only one provider in which case the facility and provider are analogous. 
5. Include a description of the types of cost analyses that will be done.

**6) Data collection during Covid-19:**

1. The authors should specify the precautions that will be taken while collecting data in the pandemic and if they anticipate any changes to the design (e.g., changes in sample sizes, types of data collected and so forth).

**7) Intervention description and theory of change:**

1. The details of the intervention are limited. Please expand.
2. I liked the multi-layered nature of the intervention from national to facility. Please make explicit as to which type of data capture will collect information on these different layers. I presume that the KIIs will capture the policy and program contexts at the national and sub-national levels; while the facility and client level information will describe the care giving process and outcomes.

Is the study design appropriate for the research question?

Yes

Is the rationale for, and objectives of, the study clearly described?

Yes

Are sufficient details of the methods provided to allow replication by others?

Partly

Are the datasets clearly presented in a useable and accessible format?

Not applicable

Reviewer Expertise:

NA

We confirm that we have read this submission and believe that we have an appropriate level of expertise to confirm that it is of an acceptable scientific standard, however we have significant reservations, as outlined above.

## References

[1] : Contraceptive uptake in post abortion care-Secondary outcomes from a randomised controlled trial, Kisumu, Kenya. *PLoS One*.2018;13(8) : 10.1371/journal.pone.0201214 e0201214 10.1371/journal.pone.0201214 30096148PMC6086397

[2] : Evaluating the impact of a quality management intervention on post-abortion contraceptive uptake in private sector clinics in western Kenya: a pre- and post-intervention study. *Reprod Health*.2018;15(1) : 10.1186/s12978-018-0452-4 10 10.1186/s12978-018-0452-4 29351797PMC5775589

# Response

**Published:** 2020-11-19

**Authors:** Elaine Charurat

**Affiliations:** Jhpiego, USA

**Notes on revision: **Thank you for giving us the opportunity to revise and resubmit the paper. We have managed to address the comments and suggestions to the best of our abilities. The corrections are in track changes on the revised manuscript. The following notes explain how we responded to each comment.

**Comment 1: **Information does exist in Kenya regarding postabortion family planning uptake. See for example:

 Mackenzius et al. (2018 ^1^).

 Wendot et al. (2018 ^2^).

**Response 1:** Thank you for those references – to clarify we did not take those into considerations for the study as they are published after the study had started but will consider them when we prepare for the manuscripts related to study results.

***Abstract***

**Comment 2: **In the Method section it’s written ‘Participants include health facilities.” Since facilities are not participants as such, please indicate if by participant the intention was to refer to health personnel.

**Response 2: **We did not mean to say that health personnel are participants, but that data is gathered from heath facilities, via pre- and post- facility assessments. However, we acknowledge that the wording did make this unclear. We have adjusted the wording in the abstract.

**Comment 3: **The method section should also include i) the duration of the operations research, ii) the targeted sample sizes for quantitative and qualitative interviews (a break up by public and private may also be useful), and iii) the time points (year) of each wave of data collection.

**Response 3: **We have added the duration of the study to this section of the abstract as well as the overall facility and structured interview sample sizes. We regret that we couldn’t include more details on the sample size and time point information in the abstract due to word limit requirements. This information is included further in the methods section of the paper.

***Introduction***

**Comment 4:** This is well written. ‘The study follows a theory of change model’. You may like to specify if the study team developed this ToC or adopted/adapted from other existing ToC. The ToC is too crowded and wondering if a simplified version with key phrases could be used instead of long sentences in the ‘inputs’, ’process’….‘impact’ blocks.

**Response 4: **The study team developed this theory of change based off of the WHO’s *Programming Strategies for Postpartum Family Planning, *as well as Jhpiego specific findings from our own PPFP Choices private sector assessments in study sites conducted prior to the introduction of the intervention in 2017; facility assessments focusing on opportunities of integrating postpartum and postabortion FP in Kenya and Indonesia conducted in 2016 by Jhpiego prior to PPFP Choices’ inception; and additional Jhpiego programming experiences within and beyond Kenya and Indonesia. This is explained on pages 5-6 of the text.

 We acknowledge that the ToC seems to be quite “wordy,” however, each word was chosen with care and we feel that by cutting the sections down any further, we will lose the nuance of what we are attempting to convey.

**Comment 5: **It’s also not very clear if the study will assess all the outcomes and also impacts. It’s worth specifying which components of the ToC the study will measure/assess.

**Response 5: **With the timing of this study, in which data collection takes place over a period of three years, we expect to be assessing the Outcomes listed in the ToC. The expectation is that if the outcomes are fully met and recommendations for scale-up of similar programs and sustainability of quality FP services are accepted, then this will, over time, lead to our expected impacts also being met. Language clarifying this expectation has been added to page 6.

***Methods***

**Comment 6: **Were the experimental and control districts matched? Are they contiguous? Is there any chance of contamination between the intervention and control districts?

**Response 6: **The study areas were matched as much as possible across a set of study criteria including number of normal deliveries, number of women attending 4+ ANC visits, number of deliveries per year and number of public and private facilities. In neither country are the study areas next to each other and there is little chance for contamination between the intervention and control areas. This has been clarified on page 11.

**Comment 7: **It was not very clear if baseline data had already been collected. It seems that interventions started in 2017 suggesting that the baseline would have had to happen prior to 2017. Please clarify.

**Response 7: **We have clarified the wording on page 10 to indicate that we are comparing intervention to control results regarding our main outcome of LARC-PM use at 6-months postpartum. Our previous wording implied that we would be comparing to a temporal baseline, which is not the case. We did however, complete baseline facility assessments and baseline KIIs with community leaders prior to intervention in early 2017.

**Comment 8: **It will be helpful if the authors could provide a diagram to describe the design as is typical of OR studies - (i.e.) the diagram should include the time points of data collection (t1,t2), the experimental and control arms (E/C), and the intervention (x).

**Response 8: **Study timeline (Figure 4) is now included in version 2 of the paper.

**Comment 9: **Glad to see that the sample size calculations accounted for clustering and the design effect.

**Response 9: **Thank you.

**Comment 10: **Please clarify if “Sample sizes were calculated to measure a change from 10% to 15% in Indonesia and from 6% to 10% in Kenya at intervention facilities over the course of the study.” Please state clearly if these are the ‘absolute’ changes in the intervention area OR, these are ‘net’ changes in the intervention area accounting for the change in the control area?

**Response 10: **As mentioned above, we have clarified the language in the sample sizes section to indicate that the change is a change in the expected outcome in the intervention area and will be compared to usual use of LARC+PM at six months postpartum rather than to any baseline measures at individual facilities.

**Comment 11:** More importantly, the prevalence rates considered for sample size calculation are of 2012 and 2014, and until the time the project began, there could be changes in these rates. Did your sample size calculation consider/factored that in and if so please specify how. 

**Response 11: **No. We used the most recent data available to us at the time to design the study. Our consultations with local government and non-profit stakeholders indicated no need to adjust our sample size calculations to reflect change in uptake. Please note that the study was designed in 2015 and approved by the IRB in 2016, so while there may have been some differences they reflect only one to three years change, rather than the more than eight years from the Indonesia DHS to today.

**Comment 12: **Similarly, for post-abortion women, the assumed 10% points change is ‘absolute’ or ‘net’ change?

**Response 12: **For postabortion women, we used a 10% net change (a decrease from 50% to 40% as stated on page 10) in the percent of women in the cohort accepting a method immediately following pregnancy loss compared to the percent of women in the cohort using a method six months later at intervention sites only. We have clarified that this is set to measure a “net” change on page 11.

**Comment 13:** Please specify the target sample size for the qualitative interviews.

**Response 13: **This information is included in Table 2, but we have clarified the language and have added the information to the narrative on page 11.

**Comment 14**: Please specify the duration of the study so that it is easier for a reader to know over what period the hypothesized change would occur.  “change from 10% to 15% in Indonesia and from 6% to 10% in Kenya at intervention facilities over the course of the study.”

**Response 14: **As noted above, we have clarified that the change is not from baseline to endline, but from control to intervention.

**Comment 15**: A strength of the proposed design is the longitudinal tracking of women.

**Response 15:** Thank you.

**Comment 16:** Include some information on what types of cost data will be collected.

**Response 16: **Kindly see response to the first reviewer on data collection for cost-related information. 

**Comment 17:** State clearly whether the respondents in the ‘multiple time points’ are cross-sectional or longitudinal cohort of women in the study design section (the mention of cohort appears much later). Also, please specify the ‘multiple’ time points in terms of year/months of surveys.

**Response 17: **Thank you, we have clarified that this data is collected longitudinally.

**Comment 18:** Was the voluntary informed consent verbal or written? In which language? Was a copy of the consent form given to them?

**Response 18: **All eligible participants were provided with written consent form in preferred language in English, Bahasa, Kimeru or Kiswahili and consent was obtained in writing with participant initials, signature or a thumbprint. A signed copy of the consent form was given to the participant for their retention. In case a participant did not wish to take the copy, both signed copies would be kept in the study folder. This has been clarified on page 14.

***Analysis***

**Comment 19: **Please add a couple of lines to indicate that the analytical approach will be a difference in difference method. This seems to be implicit but make it explicit. If so, please state clearly what would be the dependent variables and independent variables that would be adjusted for.

**Response 19: **We will not be calculating a difference-in-difference because we are not comparing baseline and endline results. Our apologies that this was not clear throughout the proposal. As previously stated, we have amended the study design section to clarify our plans. For our primary research question, we are instead intending to conduct mixed-effects logistic regression analysis to identify factors, including recruitment from an intervention facility and potentially receipt of different types of services, associated with increased odds of using a LARC+PM at six months postpartum, along with several other secondary outcomes of interest.Additional language about our analytic plan has been added to page 19.

**Comment 20: **Indicate the different levels that will be considered in the multilevel analysis.

**Response 20: **We will consider individual and facility levels. Our primary model will only include a random intercept for facility, but we may explore other models that consider additional facility-level variables. We have added language to page 19 to indicate our plans for using the random intercept.

**Comment 21: **It will also be helpful to include explicitly how public and private facilities will be compared since private sector involvement is a key component of the study.

**Response 21: **We will include a variable to measure the effect of recruitment from a public or private facility on key outcomes. This will most likely be entered into the model at the individual level, but we will explore inclusion of this variable at the facility level as well. Furthermore, we will conduct descriptive analysis comparing clients and outcomes at public and private facilities and will highlight the differences between public and private facilities in qualitative analysis.

**Comment 22: **In addition to facility level variation, there will likely be provider level variations that will need to be taken care of. Unless in certain types of facilities, there is only one provider in which case the facility and provider are analogous.

**Response 22: **Thank you for this comment. It is true that there will likely be variations in the care provided by individual providers. However, women in our study may receive care from more than one provider over the course of the study period as they transition from antenatal to labor and delivery to postpartum care. All data are collected retrospectively from clients, including shortly after delivery when recollection of the specific providers attending to a woman may be challenging. For this reason, we have chosen not to include provider-level information in any of our models. However, we have included questions about the type of care the women received and intend to explore relationships between a composite quality of care variable or variables and our primary research outcomes. A short statement on this has been added to page 19.****

**Comment 23: **Include a description of the types of cost analyses that will be done.

**Response 23: **Similar question was raised by the first reviewer, kindly see earlier response.

***Data collection during Covid-19***

**Comment 24: **The authors should specify the precautions that will be taken while collecting data in the pandemic and if they anticipate any changes to the design (e.g., changes in sample sizes, types of data collected and so forth).

**Response 24: **PPFP Choices’ data collection was completed in Indonesia before the start of the pandemic. Data collection for the first three contact points was also complete in Kenya and we were set to finish the 12 months follow-up in April 2020. On March 16, 2020, we decided to end the remaining data collection activities in Kenya. As a result, we lost 365 women at 12 months follow-up in Kenya.

***Intervention description and theory of change***

**Comment 25: **The details of the intervention are limited. Please expand.

**Response 25:** Similar question was raised by the first reviewer, kindly see earlier response.

**Comment 26: **I liked the multi-layered nature of the intervention from national to facility. Please make explicit as to which type of data capture will collect information on these different layers. I presume that the KIIs will capture the policy and program contexts at the national and sub-national levels; while the facility and client level information will describe the care giving process and outcomes.

**Response 26: **This is correct except we might also find additional information in KIIs and FGDs and will record accordingly. This has been clarified on page 10.

# Peer review: Reviewer response for version 1

**DOI:** 10.21956/gatesopenres.14338.r29282

**Published:** 2020-08-17

**Authors:** John Cleland

**Affiliations:** 1 Department of Population Health, London School of Hygiene & Tropical Medicine, London, UK

The rationale for this intervention is clear. The focus on long-acting and permanent methods (LARPMs) is welcome, as is the inclusion of private-sector facilities. The protocol is much stronger and more detailed on the research component than on the nature of the intervention itself. Perhaps, this imbalance reflects the fact that intervention activities apparently started some years ago but the consequence is that their precise nature is unspecified and impossible to replicate. For instance, is antenatal counselling in Kenya given in groups or individually? How many staff are trained in IUD/implant insertions and sterilization? Are women interested in LARPMs but unwilling to accept before discharge given an appointment to receive the method at a later date? What role, if any, do postnatal checks and child immunization visits play? What client-charges are made for provision of contraceptive devices in private facilities? Are public-sector facilities or staff given any incentives, for instance performance related bonuses, for provision of postpartum family planning?

**Overall study design:**

- If I have understood correctly, the design is a form of stepped-wedge trial, with two steps. Intervention facilities receive the package first (in 2017?) and then starting in 2020 (or whenever the covid-pandemic permits), the control facilities receive the package but no evaluation is planned for the control facilities. The sample size calculations on page 7 confused me. It is initially stated that they were calculated to detect a difference in LARPM use at 6 months postpartum between intervention and control facilities but subsequently it is stated that the size calculations were based on changes in LARPM use at 6 months between start and end in intervention facilities. On page 13, the plans for analysis imply an emphasis on intervention versus control comparisons.
- Changes in LARPM use between baseline and endline can only be estimated from facility records. If quality control checks on these records have been made, or investigators have confidence in their validity, they represent a valuable additional means of assessment and should be analysed for both intervention and control facilities. This would permit a check on the possibility that research activities in control facilities had a “Hawthorne-type” effect, resulting in an increased interest in postpartum FP among both women and staff in control facilities, and thereby complicating the intervention versus control comparison.
- The design in Kenya is much stronger than in Indonesia, both in the number of facilities selected and in the addition for Kenya of antenatal recruitment and a 12-month follow-up. There must be sound pragmatic reasons for these divergences but most are unstated. Of particular concern is the selection of only one public-sector facility in Indonesia in each arm. I doubt whether the study will be able to provide reliable indications of how postpartum FP in the public-sector can be enhanced in this country, based on only one facility in each arm.

 

**Objectives versus design:**

- The key objective is nuanced. Rather than simply assess the impact on FP uptake of the intervention package, the aim is to identify key determinants, or programming inputs, that influence FP uptake. The implication is that some components of the package add little value. As stated in the protocol, the desire is to identify what works and what does not. Ideally, a multifactorial trial design would be needed to meet this objective. Instead, I assume that answers will be sought in a subjective manner from the KIIs, IDIs and FGDs. But I wonder what the investigators have in mind. Most intervention components---staff training, supply chain, improved record keeping, counselling at different points—are indispensable and mutually reinforcing. My hunch is that the enthusiasm for the project of top facility managers will be critical but this would not be helpful for scaling-up.
- Several objectives specify cost analysis but the collection of relevant data is not mentioned in the methods. Collection of robust cost information is a huge challenge, not least because it requires staff time-use information and careful separation of implementation and research expenditure. This omission is a concern because assessment of cost-benefit is crucial for decisions about scale-up. It would be surprising if the intervention did not result in an increase in LARPMs at 6 months but the cost efficiency of the increase is uncertain.

 

**Ethical dimensions:**

- The protocol has received ethical clearance which is reassuring but I regret the absence of any discussion. Personally, I am concerned about women who are counselled for the first time and offered LARPMs immediately before or after delivery when they are vulnerable and not in the best position to give informed consent. The threat to the voluntary principle is exacerbated if staff are under any pressure or incentive to increase pre-discharge uptake and will be more common in Indonesia where many women will not have been counselled during antenatal visits. Specifically, I would regard it as unethical to offer immediate postpartum sterilization unless the woman had previously indicated a desire for a permanent method at the antenatal stage.
- A further delicate matter is follow-up of abortion cases, many of whom may wish to conceal the procedure from partners or relatives. The protocol states that the date and location of follow-up interviews will be jointly decided with participants. This is a sound tactic. It seems likely that loss to follow-up will be higher for abortion than for delivery cases.

 

**Research methods:**

- These are impressive and the mix of quantitative and qualitative data collection is welcome. Eligibility for the surveys is carefully specified and allowance for loss to follow-up seems realistic. I assume that mobile phone numbers will be routinely ascertained to facilitate follow-up. The number of KIIs and IDIs is very large (about 100 in each country). Translation, transcription, coding and analysis is very time-consuming and I hope sufficient funds have been budgeted.

 

Is the study design appropriate for the research question?

Partly

Is the rationale for, and objectives of, the study clearly described?

Yes

Are sufficient details of the methods provided to allow replication by others?

No

Are the datasets clearly presented in a useable and accessible format?

Not applicable

Reviewer Expertise:

Demography, fertility, family planning

I confirm that I have read this submission and believe that I have an appropriate level of expertise to confirm that it is of an acceptable scientific standard, however I have significant reservations, as outlined above.

# Response

**Published:** 2020-11-19

**Authors:** Elaine Charurat

**Affiliations:** Jhpiego, USA

**Notes on revision: **Thank you for giving us the opportunity to revise and resubmit the paper. We have managed to address the comments and suggestions to the best of our abilities. The corrections are in track changes on the revised manuscript. The following notes explain how we responded to each comment.

**Comment 1**: The rationale for this intervention is clear. The focus on long-acting and permanent methods (LARPMs) is welcome, as is the inclusion of private-sector facilities. The protocol is much stronger and more detailed on the research component than on the nature of the intervention itself. Perhaps, this imbalance reflects the fact that intervention activities apparently started some years ago but the consequence is that their precise nature is unspecified and impossible to replicate. For instance, is antenatal counselling in Kenya given in groups or individually? How many staff are trained in IUD/implant insertions and sterilization? Are women interested in LARPMs but unwilling to accept before discharge given an appointment to receive the method at a later date? What role, if any, do postnatal checks and child immunization visits play? What client-charges are made for provision of contraceptive devices in private facilities? Are public-sector facilities or staff given any incentives, for instance performance related bonuses, for provision of postpartum family planning?

**Response 1:** The purpose of this manuscript is the protocol itself. Therefore, we intentionally focused on the research component rather than the intervention, which is generally outlined in Figure 2a-d. We also keep country-specific intervention trackers (now as *Extended data*, DOI: https://doi.org/10.6084/m9.figshare.13318742.v1) detailing exactly what was implemented in the study areas. We hope that, once the study is completed, we will be able to make recommendations for future programming based on the study results and programmatic learnings so that others will be able to replicate and/or scale-up the effective interventions. These recommendations will be disseminated through subsequent manuscripts with study results, white papers, program briefs and other channels as appropriate. In short, the interventions were carried out from pregnancy through immediate post-pregnancy period and integrated within existing current health systems in Kenya and Indonesia.

***Overall study design***

**Comment 2**: If I have understood correctly, the design is a form of stepped-wedge trial, with two steps. Intervention facilities receive the package first (in 2017?) and then starting in 2020 (or whenever the covid-pandemic permits), the control facilities receive the package but no evaluation is planned for the control facilities. The sample size calculations on page 7 confused me. It is initially stated that they were calculated to detect a difference in LARPM use at 6 months postpartum between intervention and control facilities but subsequently it is stated that the size calculations were based on changes in LARPM use at 6 months between start and end in intervention facilities. On page 13, the plans for analysis imply an emphasis on intervention versus control comparisons.

**Response 2:** We understand why this may been seen as a stepped-wedge trial, but do not consider it to be one ourselves because intervention and control data will be simultaneously collected. Furthermore, while the control facilities will eventually receive the intervention, or a subset of the intervention package as the results of the study may recommend, this will be done outside of the study period. A stepped-wedge design has therefore not been accounted for in sample size calculations.

 We apologize if the sample size calculations were unclear. We are not measuring an increase over time but instead expect to see a higher percentage of users at 6 months postpartum in the intervention area. This is higher than the "baseline", which isn't really a baseline temporally so much as a comparison to the percent of users accepting a method under usual care.  We have added clarifying language to the third paragraph of the Sample Size section on page 10.

**Comment 3**: Changes in LARPM use between baseline and endline can only be estimated from facility records. If quality control checks on these records have been made, or investigators have confidence in their validity, they represent a valuable additional means of assessment and should be analysed for both intervention and control facilities. This would permit a check on the possibility that research activities in control facilities had a “Hawthorne-type” effect, resulting in an increased interest in postpartum FP among both women and staff in control facilities, and thereby complicating the intervention versus control comparison.

**Response 3: **As noted above, we have revised the Sample Size section to clarify that we are comparing, not baseline to endline, but control facilities to intervention facilities among a cohort of postpartum women.

**Comment 4: **The design in Kenya is much stronger than in Indonesia, both in the number of facilities selected and in the addition for Kenya of antenatal recruitment and a 12-month follow-up. There must be sound pragmatic reasons for these divergences but most are unstated. Of particular concern is the selection of only one public-sector facility in Indonesia in each arm. I doubt whether the study will be able to provide reliable indications of how postpartum FP in the public-sector can be enhanced in this country, based on only one facility in each arm.

**Response 4:** We agree with the reviewer comments about the limitations of private sector findings in Indonesia (we have 3 public sector facilities and 1 private sector facility per arm), and acknowledge that the study would have been stronger had we recruited equal sized samples from each facility. However, actual site selection was done based on programs needs and convenience given the existing relationships and presence of the MyChoice program in Indonesia. Within MyChoice and PPFP Choices study districts, a smaller number of facilities met the study criteria to receive the interventions, but these facilities, especially qualifying private sector facilities, each experience a higher volume of patients per month than the Kenyan facilities. Use of these facilities therefore allowed us to meet the desired public and private participant sample size, but unfortunately resulted in inclusion of only one large private facility per study arm. All analysis takes this variety into consideration and controls, where possible and reasonable, for facility level differences. We are planning a mixed-effects model with a random intercept for facility and have included this information under our data entry and analysis section of the updated version this publication. 

 We agree that it would have been ideal to complete ANC and 12-month interviews in both Kenya and Indonesia. In Indonesia we chose to recruit women from labor and delivery (L&D) rather than ANC because women are known to frequently change facilities and providers from ANC to L&D. As this would have made it more difficult to track women through the system and ensure that they continued to receive care from intervention facilities if recruited from one, we chose to simplify the process and recruit women after delivery. This is outlined on pages 12 and 13. We were also, unfortunately, unable to secure additional funding and IRB approval for the 12-month interview in Indonesia until after many of the Indonesian participants passed 12-months postpartum. As this would have led to a sample size too small for our planned analyses, we chose to omit the 12-month interview from the plan in Indonesia. We have added language to indicate why the 12-month interview as not conducted in Indonesia to page 13.

***Objectives versus design***

**Comment 5: **The key objective is nuanced. Rather than simply assess the impact on FP uptake of the intervention package, the aim is to identify key determinants, or programming inputs, that influence FP uptake. The implication is that some components of the package add little value. As stated in the protocol, the desire is to identify what works and what does not. Ideally, a multifactorial trial design would be needed to meet this objective. Instead, I assume that answers will be sought in a subjective manner from the KIIs, IDIs and FGDs. But I wonder what the investigators have in mind. Most intervention components---staff training, supply chain, improved record keeping, counselling at different points—are indispensable and mutually reinforcing. My hunch is that the enthusiasm for the project of top facility managers will be critical but this would not be helpful for scaling-up.

**Response 5: **We agree with the reviewer’s point on overall the study’s main goal is to assess the impact on family planning uptake at six-month postpartum. Based on existing programming experience and literatures, we also understand PPFP interventions requires all relevant components but are hoping to identify most crucial elements for future programming consideration. We also agree with the reviewer’s point on facility managers as they play critical role as they are encouraged and empowered to take ownership during implementation. Additionally, while data from client interviews will give us direct associations on key factors such as women’s demographic characteristics, fertility intention, counseling, method availability etc, we are hoping the qualitative results from KIIs, IDIs and FGDs will reveal facilitators and barriers which may not be covered during client exit and follow-up interviews.

****

**Comment 6: **Several objectives specify cost analysis but the collection of relevant data is not mentioned in the methods. Collection of robust cost information is a huge challenge, not least because it requires staff time-use information and careful separation of implementation and research expenditure. This omission is a concern because assessment of cost-benefit is crucial for decisions about scale-up. It would be surprising if the intervention did not result in an increase in LARPMs at 6 months but the cost efficiency of the increase is uncertain.

**Response 6:** We initially intended to conduct a more robust cost analysis. However, we were unable to collect the needed cost elements due to budget constraints and a prioritization of other learning objectives. Instead, we are hoping to gain insights into and examine those cost related topics from qualitative results, as it is an important consideration for scale-up. We have updated Table 1’s title from PPFP Choices study themes and key questions to “PPFP Choices study themes and key questions to be explored” to avoid further confusion.

***Ethical dimensions***

**Comment 7: **The protocol has received ethical clearance which is reassuring but I regret the absence of any discussion. Personally, I am concerned about women who are counselled for the first time and offered LARPMs immediately before or after delivery when they are vulnerable and not in the best position to give informed consent. The threat to the voluntary principle is exacerbated if staff are under any pressure or incentive to increase pre-discharge uptake and will be more common in Indonesia where many women will not have been counselled during antenatal visits. Specifically, I would regard it as unethical to offer immediate postpartum sterilization unless the woman had previously indicated a desire for a permanent method at the antenatal stage.

**Response 7:** Thank you for raising this point. We wholeheartedly agree with the reviewer’s concerns and apologize if this protocol appears to promote any type of coercive behavior. The interventions are not described in detail in this paper because it focuses on the protocol itself, but all interventions are carried out to ensure postpartum and postabortion women have access to high-quality, safe and effective voluntary family planning if and when they choose. Although immediate postpartum sterilization is uncommon in study settings, we expect that the trained staff are able to attain and carry out quality family planning counselling and service delivery for all methods with a hope that women are afforded more opportunity to make informed decisions about their PPFP needs. In fact, a review of initial data from Indonesia (not presented in this paper), where postpartum sterilization is more common, found that the intervention decreased the percent of women receiving tubal ligations prior to discharge and diversified the method mix among PPFP acceptors at intervention sites. As part of quality assurance, the study team have kept a strict eye on family planning uptake and have been instructed to report any signs of coercion through regular monitoring of study data and quarterly facility visits. We also plan to validate quality of family planning counselling and informed choice via qualitative data collection. 

**Comment 8: **A further delicate matter is follow-up of abortion cases, many of whom may wish to conceal the procedure from partners or relatives. The protocol states that the date and location of follow-up interviews will be jointly decided with participants. This is a sound tactic. It seems likely that loss to follow-up will be higher for abortion than for delivery cases.

**Response 8:** We agree that follow-up of post-abortion cases is a very delicate matter, whether because the participant may not want to think and/or share her experience with others, or because she might be afraid of stigma. We have built a 20% loss to follow up between interview 1 and interview 2 into the sample size calculations, which is more than the expected loss to follow up for postpartum participants.

***Research methods***

**Comment 9: **These are impressive and the mix of quantitative and qualitative data collection is welcome. Eligibility for the surveys is carefully specified and allowance for loss to follow-up seems realistic. I assume that mobile phone numbers will be routinely ascertained to facilitate follow-up. The number of KIIs and IDIs is very large (about 100 in each country). Translation, transcription, coding and analysis is very time-consuming and I hope sufficient funds have been budgeted.

**Response 9:** Thank you. As suggested, we gather mobile phone numbers and physical addresses of all participants and have a multi-stage plan to connect with women prior to their next follow-up interview. Using this plan, our loss to follow-up has been lower than what was built into our study design. We agree that the number of qualitative interviews is large, but felt it was necessary for adequate mixed methods research. We have a good team working on our qualitative analysis using Atlas.TI. As of now, we have completed all Postabortion care analyses and are in the process of completing Postpartum and Key Informant interview analyses.
